# Diagnosis, treatment, and follow-up of heart failure patients by general practitioners: A Delphi consensus statement

**DOI:** 10.1371/journal.pone.0244485

**Published:** 2020-12-31

**Authors:** Caroline Verhestraeten, Gijs Weijers, Daphne Debleu, Agnieszka Ciarka, Marc Goethals, Steven Droogmans, Michael Maris

**Affiliations:** 1 Novartis Pharma nv-sa, Vilvoorde, Belgium; 2 Department of Cardiology, Centre Hospitalier Bois de l’Abbaye, Seraing, Belgium; 3 Department of Cardiovascular Diseases, University Hospitals Leuven, Leuven, Belgium; 4 Katholieke Universiteit Leuven, Leuven, Belgium; 5 Department of Cardiology, OLV Hospital Aalst, Aalst, Belgium; 6 Department of Cardiology, Centrum Voor Hart-en Vaatziekten, Jette, Belgium; Maastricht University Medical Center, NETHERLANDS

## Abstract

**Aims:**

Creation of an algorithm that includes the most important parameters (history, clinical parameters, and anamnesis) that can be linked to heart failure, helping general practitioners in recognizing heart failure in an early stage and in a better follow-up of the patients.

**Methods and results:**

The algorithm was created using a consensus-based Delphi panel technique with fifteen general practitioners and seven cardiologists from Belgium. The method comprises three iterations with general statements on diagnosis, referral and treatment, and follow-up. Consensus was obtained for the majority of statements related to diagnosis, referral, and follow-up, whereas a lack of consensus was seen for treatment statements. Based on the statements with good and perfect consensus, an algorithm for general practitioners was assembled, helping them in diagnoses and follow-up of heart failure patients. The diagnosis should be based on three essential pillars, i.e. medical history, anamnesis and clinical examination. In case of suspected heart failure, blood analysis, including the measurement of NT-proBNP levels, can already be performed by the general practitioner followed by referral to the cardiologist who is then responsible for proper diagnosis and initiation of treatment. Afterwards, a multidisciplinary health care process between the cardiologist and the general practitioner is crucial with an important role for the general practitioner who has a key role in the up-titration of heart failure medication, down-titration of the dose of diuretics and to assure drug compliance.

**Conclusions:**

Based on the consensus levels of statements in a Delphi panel setting, an algorithm is created to help general practitioners in the diagnosis and follow-up of heart failure patients.

## Introduction

Heart failure (HF) is a complex syndrome that is characterized by clinical manifestations, such as breathlessness, ankle swelling, and fatigue and typically accompanied by signs, such as elevated jugular venous pressure, pulmonary crackles and peripheral edema. These symptoms and signs are caused by structural and functional impairments resulting in reduced cardiac output or elevated ventricular filling pressure at rest or during stress [1].

HF will become one of the most common diseases for the elderly since approximately 26 million adults are currently living with HF worldwide, a number that is expected to rise towards 2030 [2]. Data from registries currently demonstrates 1–2% prevalence of HF that increases to 10% and more in people aged 70 and over. Additionally, the prevalence of HF will increase further over time due to aging of the population and expanding occurrence of comorbidities [2, 3].

Importantly, each year, around 20% of all HF patients are hospitalized, which makes HF a leading cause of hospitalization, associated with a high economic burden on our health systems. It was calculated that the healthcare cost for HF patients accounts for 1–3% of the total healthcare expenditure in North and Latin America, as well as in Europe [2].

The general practitioners (GPs) play an essential role in the management of HF as the first clinical presentation usually takes place in the general practice setting, and as they are responsible for the daily follow up of chronic HF patients [1]. Nevertheless, a significant amount of GPs have difficulties with diagnosing HF due to the unspecific nature of signs and symptoms of HF [4–6]. Studies mapping the barriers affecting the diagnostic process for GPs showed that GPs were unfamiliar with the natural history of HF, lacked the tools (e.g. cardiac ultrasound and N-terminal pro B-type natriuretic peptide (NT-proBNP)) to diagnose and manage HF and they were not fully aware of relevant research evidence and guidelines. Also, the GPs’ need for education was expressed, as well as the importance of a more chronic care approach of HF [7–9]. As a result, there is an underdiagnosis, as shown by the high prevalence rates of unrecognized HF (constituting up to 80% of all HF cases) in high-risk community populations, e.g. older people with breathlessness, type 2 diabetes or COPD from primary care. When these patients present themselves to the GP, symptoms that could suggest HF may not be recognized as such or may be confused with other diagnoses, and might not be reported by the patients either [10]. Smeets *et al*. concluded that a paradigm shift is needed towards an earlier and more comprehensive risk assessment with, among others, access to natriuretic peptide testing and convincing GPs of the added value of a validated HF diagnosis [8, 9].

Even though, guidelines on heart failure exists, it is clear that there is an urgent need for a more practical and easy to use algorithm, based on non-invasive, non-radiographic parameters that can be implemented in the GP’s daily practice, to recognize potential HF patients in an early stage leading to fast and early referral to the cardiologist. Therefore the objective of this project was to create a hands-on-algorithm, starting from clinical anamnesis to guide GPs in the diagnosis, referral and treatment, and follow-up of HF patients based on non-invasive parameters, using the Delphi technique for a consensus-based approach.

## Materials and methods

### Design

The Delphi technique is a widely used method for achieving a consensus by using a series of questionnaires to collect real-world knowledge from a small panel of experts (between 10 and 20 respondents) in a specific topic area. The main characteristic of the classic Delphi technique is the feedback process in which the responses of the questionnaire of the first round generate qualitative data and are then returned to the group during the next round in the form of statistical summaries. This feedback process allows and encourages the participants to reassess their initial judgement provided in previous rounds. A consensus is reached after analyzing multiple iterative questionnaires [11]. At least three iterations are needed for a valid classic Delphi process [12]. In this study, the classic Delphi technique was selected as a qualitative questionnaire was sent to the panel and used to create the questions of the second iteration. An important advantage of the classic Delphi technique is anonymity, which can reduce the effects of dominant individuals or group pressure when using group-based processes to collect and analyze information. This anonymity results from the absence of direct interaction between participants during the entire process [11, 12].

#### Expert panel members

The survey is conducted with a panel of two types of healthcare professionals (HCPs), i.e. GPs and cardiologists. A geographically representative sample of 15 GPs and 7 cardiologists was selected. Selection of GPs was based on HF interest, and availability to participate whereas the selection of the cardiologists was based on a particular interest in HF, previously demonstrated collaboration with GPs, and availability to participate. Each potential panel member was provided with full information on the survey, the Delphi technique and the timing of their expected involvement. Creation of an algorithm is based on the knowledge of experts, therefore all panel members had a high interest in HF and were called “experts in the field of HF”. High interest for GPs was self-reported as GP stated to have a high number of HF patients and a particular interest in HF management. For SP, they reported of being a HF specialist (also sepf-reported as there is no official recognition of this sub-specialization) and being employed in HF clinics. The two groups of experts, i.e. GPs and cardiologists, were assessed separately, with the GPs as most important and the cardiologists as a benchmark. The reason for this was the fact that an algorithm will be created for GPs by GPs, meaning that the input of the GPs has the highest value and importance. As a result, the group of GPs was chosen to be larger than the groups of cardiologists and a different level of consent for GPs and cardiologists was chosen (cfr. Determining consensus).

### Delphi rounds

The survey comprises 3 iterations, needed for the validity of the Delphi process [12]. The first iteration consisted of open-ended questions, whereas the next two iterations were closed-ended questions. By using open-ended questions in the first iteration, insights were obtained and participants were not limited or biased by predefined answers. Cardiologists were asked to respond to the questions from a GP point of view since the algorithm will be designed for use in the GP’s office. The first round was developed in SurveyGizmo and a link was sent to the panel via email. Participants had 2 weeks to complete the survey and reminders were sent after 1 week. After the first round, responses were analyzed and used to create the closed-ended questions of round 2 using an 11-Point Likert response scale. The second round was created in SurveyGizmo as well and sent to the participants via email. Participants had to indicate on an 11-Point Likert response scale how much they agreed with the statements within 2 weeks after sending the second round. After 1 week, reminders were sent. After the second round, all questions were analyzed and the responses on each question were presented as median ± interquartile range (IQR). Participants were asked during round 3 to confirm or modify the previous answers they provided in round 2 based on the given information in order to increase the consensus. Each question was included with an open field for additional explanations. Besides their own answers to the questions of round 2, the participants were also provided with the median and IQR of the answers of both GPs and cardiologists. After 3 rounds, the overall consensus was assessed.

### Questionnaire

The questions provided in the first iteration were developed based on literature review and the European Society of Cardiology (ESC) guidelines. A first version of statements was developed and categorized into three groups: questiosn related to diagnosis, referral and treatment, and follow-up of HF patients. These statements were first discussed during face-to-face interviews with 5 dedicated HF cardiologists for relevance, completeness and clarity. Their comments and suggestions were implemented to create the final questionnaire for the first Delphi round. The questionnaire for the first Delphi round can be found in S1 and S2 Figs. both in the original language (Dutch/French) as in English in S3 Fig.

### Determining consensus

An 11-Point Likert response scale was used for participants to rate their level of agreement with each statement. This scale is a 0 to 10 scale, where 5 means no difference. The level of consensus was defined as perfect, good, some and no consensus, as outlined in Table 1. The percentages linked to the different levels of consensus were pre-defined and based on literature search [11, 13]. While there is no agreement on the best approach, different levels of agreement is the most commonly used and hence was adopted. In addition, there is no accepted, set standard for the percentage of consensus but 70% is commonly reported in the literature [11, 14]. We decided to start from that percentage for the “good consensus” level for cardiologists but made it slightly stricter for the GPs. The reason for this different percentage between GPs and cardiologists, was, since the number of participating GPs was twice as high as the number of participating cardiologists (15 versus 7), a different number of each is required for the set consensus percentage. Almost all cardiologists should agree to get a consensus rate of 80% (= 6/7) while for the GPs a 80% consensus is reached if 12 GPs (out of 15) would agree. Therefore, we decided to apply different levels of good consensus for GPs and cardiologists, as outlined in Table 1. We also decided to subdivide the top consensus levels (perfect and very good) to have more insights in the results.

**Table 1:** Definition of levels of consensus.

Level of consensus^a^	GPs (pointing ≥ 8 on 11-Point Likert scale)	Cardiologists (pointing ≥ 8 on 11-Point Likert scale)
Perfect consensus	100%	100%
Very good consensus	90%	90%
Good consensus	≥ 80%	≥ 70%
Some consensus	≥ 60%	≥ 60%
No consensus	all other cases	all other cases

^a^The share of participating GPs is twice as high as the share of participating cardiologists (15 versus 7), therefore the cut-off rates for the different levels of consent were lower for the cardiologists.

### Statistical analysis

Completed questionnaires were included in data analysis. The data were analyzed using descriptive statistics and presented as median (Q2), quartiles, and interquartile range (Q1–Q3).

Differences between cardiologists and GPs were analyzed using a two-sided non-parametric Mann-Whitney U test. All statistical analyses were performed using GraphPad Prism version 8.1.2 for Windows (GraphPad Software, La Jolla California USA). P-values below 0.05 were considered as being statistically significant.

### Algorithm creation

An algorithm for the diagnosis, referral & treatment, and follow-up of HF patients was created based on statements that achieved consensus after the third iteration. The statements with perfect to good consensus formed the body of the algorithm. The statements with some consensus of the cardiologists were also included in the algorithm as “tips” for the GPs. The algorithm created based on the Delphi questionnaires results were further discussed, evaluated and fine-tuned during a round table discussion with 4 new, independent cardiologists and characterized as HF specialists in their hospital. These 4 cardiologists had again a major interest in HF. This was also specified as being a HF specialist (self-reported as there is no official recognition of this sub-specialization) and being employed in HF clinic.

## Results

### Expert panel member participation

The participation rate of panel members is given in Table 2. The first round has been completed in the respected timeframe of 2 weeks by 6 (86%) of the cardiologists and by 14 (93%) of the GPs. As the first round contained open-ended questions and insights were used to create the statements in the second iteration, round 2 has been sent out to all the physicians, even to the one GP and one cardiologist who did not respond in the first round. Therefore the response rate for round 2 was 100% for both the GPs and cardiologists, whereas 7 cardiologists (100%) and 13 (87%) GPs completed the third round of the Delphi technique.

**Table 2:** Fully completed questionnaires in the three iterations taken into account for analysis.

Panel	Round 1	Round 2	Round 3
Cardiologists	6/7 (86%)	7/7 (100%)	7/7 (100%)
GPs	14/15 (93%)	15/15 (100%)	13/15 (87%)
Total	20/22 (91%)	22/22 (100%)	20/22 (91%)

### Statement consensus

Statements were categorized into 3 groups, i.e. diagnosis, referral and treatment, and follow-up, consisting of 8, 10 and 18 statements, respectively. After round 3, a perfect consensus for all HCPs was obtained for 63%, 20% and 56% for statements regarding diagnosis, referral and treatment, and follow-up, respectively. More detailed, consensus reached at the GP level was 63%, 20% and 33% for the 3 groups respectively, while the level of agreement for cardiologists was higher in all 3 groups (88%, 40%, 78%, respectively). Consensus was not obtained by the cardiologists nor by the GPs for 14 statements, which corresponds to 39% (14/36) and is mainly due to a lack of consensus of the treatment statements.

#### Diagnosis

A good to perfect consensus was reached by both cardiologists and GPs regarding the importance of dyspnea, orthopnea, fatigue, weight gain, the specific medical history during the anamnesis for suspicion of HF. In line, the presence of edema and lung crepitations during the clinical examination is of utmost importance, whereas inspection of the ankles and feet, blood pressure measurement, increased heartbeat and weight gain should be taken into account carefully when one suspects HF. Statements related to the importance to measure certain lab parameters (eg. kidney and liver function); as well as the importance of NT-proBNP as a tool to rule out HF reached good consensus at the cardiologists’ level, but reached only some or no consensus at the GP level (Table 3).

**Table 3:** Number of responses and consensus rate reached after the third round based on statements regarding to diagnosis.

		Card		GPs		Card	GPs	Consensus		MWU
Statement		M	IQR	M	IQR	≥ 8	≥ 8	Card	GPs	p-value
1	If dyspnea, breathlessness after exercise and ankle edema are present during anamnesis, I will suspect heart failure	10	1,5	10	1,8	100%	93%	P	V	NS
2	If orthopnea, fatigue, and weight gaining are present during anamnesis, my suspicion of heart failure will increase	10	0,5	9	1,8	100%	86%	P	G	NS
3	If cardiac history, arterial hypertension and/or diabetes are present in the medical history of the patient, heart failure will be more likely	10	0	9,5	1	100%	93%	P	V	NS
4	If obesity, chronic kidney insufficiency and/or COPD are present in the medical history of the patient, my suspicion of heart failure will increase	7	2	8,5	4,8	43%	57%	N	N	NS
5	To increase my suspicion of heart failure, I will perform heart and lung auscultation, inspection of the ankles and feet, blood pressure measurement, heartbeat, and weight gain	10	0,5	10	0,8	100%	93%	P	V	NS
6	The two most important abnormalities in the clinical examination that make heart failure the most plausible diagnosis are edema and lung crepitations	9	1,5	10	1	71%	86%	G	G	NS
7	In case of suspicion of heart failure, evaluation of the blood parameters hematology, liver function, kidney function, transferrin saturation, ferritin, creatinine, and ionogram is recommended	10	1	8	2	86%	57%	G	N	<0.05
8	To confirm heart failure, evaluation of NT-proBNP is essential	8	4	8	3,5	71%	64%	G	S	NS

Card: cardiologists; GPs: general practitioners; M: median; ICR: interquartile range; P: perfect consensus; V: very good consensus; G: good consensus; S: some consensus; N: no consensus; MWU: Mann-Whitney U test. NS: non-significant.

#### Referral and treatment

Good to perfect consensus by cardiologists and GPs was reached regarding the importance to send a patient with dyspnea, orthopnea to the cardiologist when the GP suspects HF. While there was a perfect consensus at the cardiologist level to refer a patient with a suspicion of HF and edema from the GP to the cardiologists’ office, there was only some consensus at the GP level. The cardiologists reached a good consensus regarding the initiation of diuretics and angiotensin-converting enzyme inhibitors (ACEI) by GPs in anticipation of the patient's appointment with the cardiologist when the patient’s main complaints are breathlessness and lung problems. The GPs only reached some consensus for this statement. All other statements related to the initiation of treatment at the GP office did not reach any consensus nor by the cardiologist, nor by the GPs (Table 4).

**Table 4:** Number of responses and consensus rate reached after the third round based on statements regarding to referral and treatment.

		Card		GPs		Card	GPs	Consensus		MWU
Statements		M	IQR	M	IQR	≥ 8	≥ 8	Card	GPs	p-value
9	I will send a patient with dyspnea to the cardiologist when I suspect heart failure	10	0	10	0,8	100%	86%	P	G	NS
10	I will send a patient with orthopnea to the cardiologist when I suspect heart failure	10	0	10	0	100%	93%	P	V	NS
11	I will send a patient with edema to the cardiologist when I suspect heart failure	10	0	9,5	3,8	100%	64%	P	S	NS
12	Patient in whom I suspect heart failure and with the main complaint of breathlessness, I will initiate diuretics and ACEI in anticipation of the patient's appointment with the cardiologist	7,5	2	8,5	2,8	29%	57%	N	N	NS
13	Patient in whom I suspect heart failure and with main complaints of breathlessness and edema, I will initiate diuretics in anticipation of the patient's appointment with the cardiologist	9,5	3	7,5	3,5	57%	50%	N	N	NS
14	Patient in whom I suspect heart failure and with main complaints of breathlessness and cardiac history, I will initiate diuretics and ACEI in anticipation of the patient's appointment with the cardiologist	9	3	8,5	3,8	57%	64%	N	S	NS
15	Patient in whom I suspect heart failure and with main complaints of breathlessness and lung problems, I will initiate diuretics and ACEI in anticipation of the patient's appointment with the cardiologist	5	1,5	7,5	2,8	0%	50%	N	N	<0.01
16	Patient in whom I suspect heart failure and with main complaints of breathlessness and lung problems, I will initiate diuretics and ACEI in anticipation of the patient's appointment with the cardiologist	10	2	9	4,8	86%	64%	G	S	NS
17	Patient in whom I suspect heart failure and with main complaints of breathlessness and known diabetes, I will initiate diuretics and ACEI in anticipation of the patient's appointment with the cardiologist	7	2	7,5	4,8	29%	50%	N	N	NS
18	Patient in whom I suspect heart failure and with main complaints of breathlessness and poor kidney function, I will initiate diuretics in anticipation of the patient's appointment with the cardiologist	8	4,5	5,5	4,3	43%	14%	N	N	NS

Card: cardiologists; GPs: general practitioners; M: median; ICR: interquartile range; P: perfect consensus; V: very good consensus; G: good consensus; S: some consensus; N: no consensus; MWU: Mann-Whitney U test. NS: non-significant.

#### Follow-up

Regarding the follow-up of the HFpatient, good to perfect consensus by the cardiologists and the GPs is reached that the GP should do heart and lung auscultation, measure heart rate and blood pressure and evaluate body weight and presence of edema, whether or not the patients expresses complaints. Moreover, a control blood test measuring kidney and liver function, together with a complete ionogram reached consensus. Whereas there is perfect consensus at the cardiologist level that a GP should reduce diuretics and uptitrate beta-blockers and renin-angiotensin-aldosterone system (RAAS) inhibitors in HF patients that are feeling well and should not change the medication in a HFpatient with asymptomatic low blood pressure, there is no consensus regarding these statements at the GP level. In line, whereas the cardiologists aim for a quarterly blood test in a diagnosed HFpatient, the GPs do not agree (Table 5).

**Table 5:** Number of responses and consensus rate reached after the third round based on statements regarding to follow-up.

		Card		GPs		Card	GPs	Consensus		MWU
Statements		M	IQR	M	IQR	≥ 8	≥ 8	Card	GPs	p-value
19	When a heart failure patient comes for consultation after his appointment with the cardiologists and indicates that he/she is feeling well, my control examination will consist of heart and lung auscultation, measurement of heart rate and blood pressure and evaluation of edema and weight	10	0,5	10	0,8	100%	86%	P	G	NS
20	When a heart failure patient comes for consultation after his appointment with the cardiologists and indicates that he/she is feeling well, I will adjust the medication based on blood pressure, heart rate and kidney function	10	0,5	7	4	86%	36%	G	N	<0.01
21	When a heart failure patient comes for consultation after his appointment with the cardiologists and indicates that he/she is feeling well, I will reduce diuretics and uptitrate beta-blockers and RAAS inhibitors	9	3,5	4	5,5	71%	7%	G	N	<0.01
22	When a heart failure patient comes for consultation after his appointment with the cardiologists and indicates that he/she is short of breath, my control examination will at least consist of blood pressure measurement and lung auscultation	8	2	10	0,8	86%	86%	G	G	NS
23	Based on the deviations from previous examinations of question 19, I will increase diuretics in a patient with shortness of breath	8	1	8	3,3	100%	71%	P	S	NS
24	When a heart failure patient comes again for consultation after his appointment with the cardiologists and indicates that he/she has again edema, my control examination will consist of blood pressure measurement, heart rate and kidney function	9	1,5	8,5	2,8	100%	71%	P	S	NS
25	Based on the deviations from previous examinations of question 19, I will increase diuretics in a patient with edema	9	0,5	8	2,8	100%	57%	P	N	NS
26	When a heart failure patient comes again for consultation after his appointment with the cardiologists and indicates that he/she has edema again, my control examination will consist of blood pressure measurement, heart rate and kidney function	10	1,5	9,5	1,8	100%	79%	P	S	NS
27	Based on the deviations from previous studies of question 19, I will reduce diuretics, RAAS inhibitors, and beta-blockers in a heart failure patient	7	2,5	8	2,8	14%	57%	N	N	NS
28	When a heart failure patient comes again for consultation after his appointment with the cardiologists and indicates that he/she has asymptomatic low blood pressure, my control examination will consist of a new blood pressure measurement	10	0,5	10	0,8	86%	86%	G	G	NS
29	I will not change the medication in a heart failure patient with asymptomatic low blood pressure	10	0	10	1	100%	79%	P	S	NS
30	When a heart failure patient comes again for consultation after his appointment with the cardiologists and indicates that he/she has a symptomatic low blood pressure, my control examination will consist of a new blood pressure measurement and evaluation of the medication	10	0,5	10	0	100%	93%	P	V	NS
31	Based on the examinations of question 19, I will reduce diuretics, RAAS inhibitors, and beta-blockers in a heart failure patient with asymptomatic low blood pressure	9	4	6,5	3,8	57%	43%	N	N	NS
32	When a heart failure patient comes again for consultation after his appointment with the cardiologists and indicates that he/she is tired, my control examination will consist of blood pressure measurement, heart rate, lung auscultation and blood parameters measurement of at least HCT, Fe (ferritin and transferrin saturation), ionogram and kidney function	9	2	10	1,8	86%	86%	G	G	NS
33	Based on the examinations of question 19, I will increase diuretics and reduce RAAS inhibitors and beta-blockers in a heart failure patient with fatigue	4	5,5	4,5	4,5	14%	21%	N	N	NS
34	In case of a diagnosed heart failure patient, I will immediately perform a blood test if the patient shows worsening of symptoms and/or edema	8	3,5	6,5	5,5	57%	36%	N	N	NS
35	I will perform a quarterly blood test in a diagnosed heart failure patient	9	2	5,5	4,3	71%	29%	G	N	NS
36	For a control blood test in a diagnosed heart failure patient, I will at least check kidney function, ionogram, liver function and ferritin/transferrin saturation	9	2	10	1,8	86%	86%	G	G	NS

Card: cardiologists; GPs: general practitioners; M: median; ICR: interquartile range; P: perfect consensus; V: very good consensus; G: good consensus; S: some consensus; N: no consensus; MWU: Mann-Whitney U test. NS: non-significant.

### Algorithm

**Fig 1:**
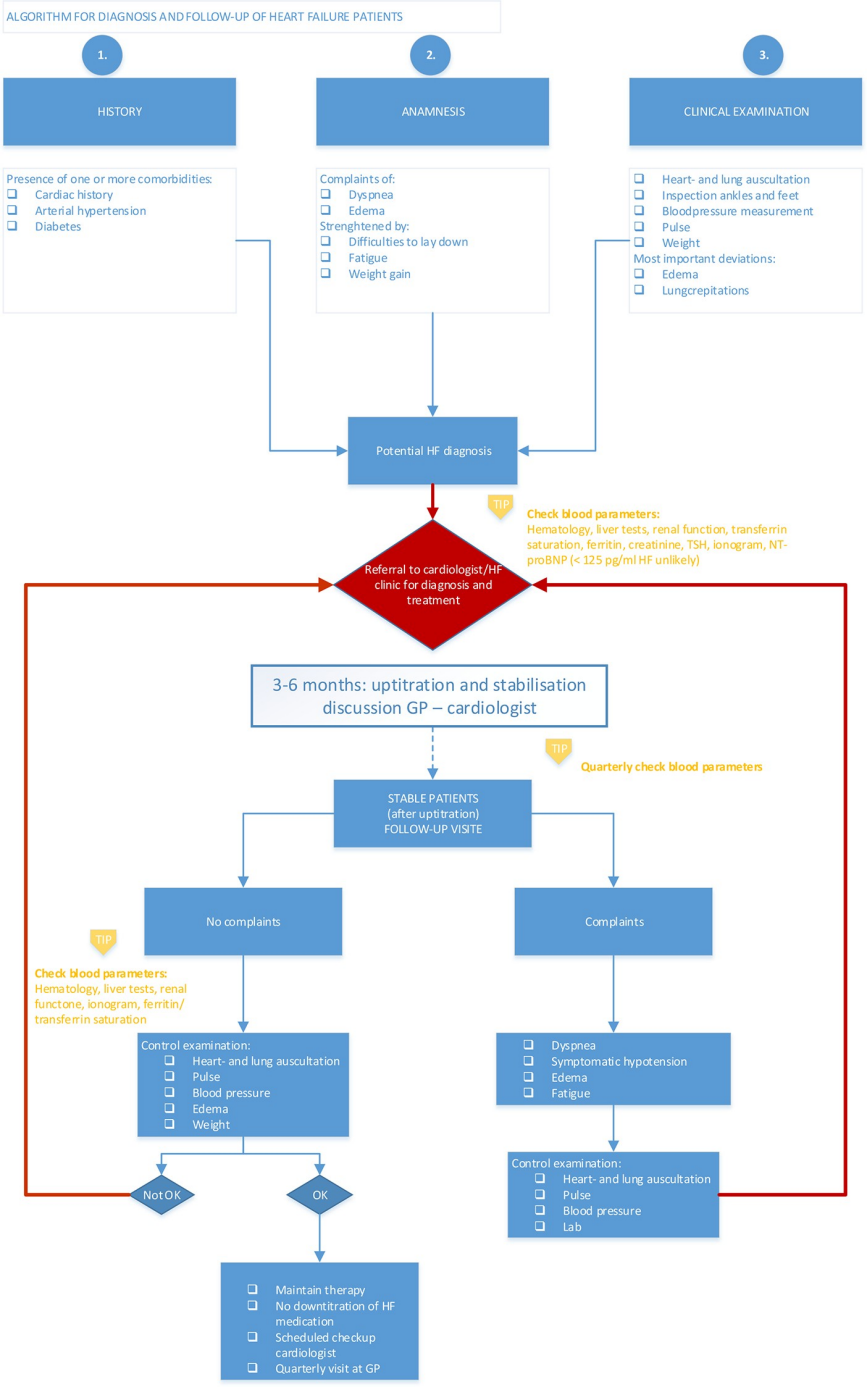
Algorithm for diagnosis and follow-up of HF patients.

An algorithm for the diagnosis, referral and treatment, and follow-up of HF patients was created based on statements that achieved consensus after the third iteration. The statements with perfect to good consensus formed the body of the algorithm. The statements with some consensus reached by the cardiologists were also included in the algorithm as “tips” for the GPs, e.g. the measurement of several blood parameters, such as such as ferritin, creatinine, liver and kidney function, etc. During a round table discussion with cardiologists with a specific interest in HF, the algorithm was fine-tuned based on the feedback from their clinical practice and the final algorithm can be found in Fig 1.

The algorithm consists of two major blocks, i.e. diagnosis and follow-up. Regarding diagnosis, medical history, anamnesis, and clinical examination are taken into account. When the GP rules out a potential diagnosis of HF, blood parameters, including NT-proBNP, can be performed, followed by referral to the cardiologist for proper diagnosis and treatment initiation. In the first instance, the cardiologist would be responsible for a proper uptitration and stabilization of the disease, followed by quarterly follow-up visits by the GP. In case the HF patients have specific complaints, the GP should perform a thorough control examination, including blood analysis, followed by referral to the cardiologist. In case of no complaints, the GP will perform a control examination and ensures that the HF medication is not stopped or downtitrated and that a yearly checkup visit with the cardiologist is scheduled.

## Discussion

GPs play a key role in HF management as HF patients present themselves with the first signs and symptoms at the GP. If a GP can recognize these signs and symptoms in a timely manner and assure referral to a cardiologist in time, treatment can start early and the disease can be delayed which leads to an enormous reduction in HF hospitalization and mortality [15]. GPs find it difficult to correctly diagnose HF and the non-specific symptoms or the overlap of symptoms and signs with comorbidities make it even more challenging [16]. These barriers lead to under- and over-diagnosis of HF [15, 17]. A consensus was reached between cardiologists and GPs regarding diagnosis and follow-up, although the consensus obtained for follow-up was mainly related to the monitoring of parameters during control examinations and not related to initiation and changing of medication. The cardiologists stated that they did not expect from the GP to initiate or change HF medication and this was confirmed by the GPs stating that this is the role of the cardiologist as they do not feel comfortable initiating HF medication [18].

Regarding the diagnosis, there are three essential pillars, i.e. medical history, anamnesis and clinical examination that should be evaluated by the GP. A cardiac history (myocardial infarction, coronary artery disease), hypertension and type 2 diabetes were included as the major comorbidities associated with an increased risk for HF. This is in line with a previous study identifying clinical factors associated with risk of incident HF as the strongest independent associations for incident HF were coronary artery disease, diabetes mellitus; and hypertension [19]. Dyspnea and edema were included in the algorithm as the most important clinical signs, further strengthened by fatigue, orthopnea, and weight gain, whereas lung crepitations and edema again were included as the most important deviations based on clinical examination. All these predictive signs are in line with a Belgian study by Devroey et al. [20]. This study prospectively collected data during a 2-year period by a Belgian network of sentinel practices. All adult patients with known HF, for which the diagnosis of HF was clincally suspected for the first time, were registered. 754 patients with suspicion of HF were recorded of which 74% got a confirmatory diagnosis of HF [20]. From a logistic regression, breathlessness on exercise, limitations of physical activity, and orthopnea were the symptoms most associated with HF [20]. When looking into our algorithm, dyspnea and orthopnea were also included. Regarding clinical signs, we also included pulmonary rales and edema in the algorithm, being the two clinical signs that were most associated with HF according to the study of Devroey et al. [20]. It is mentioned that several symptoms and clinical signs have good specificity but sensitivity is only good for breathlessness on exercise, pulmonary rales, peripheral edema, and limitation of physical activity, as some key symptoms, such as tiredness, fatigue, and breathlessness are very nonspecific among elderly and obese patients [20]. Nevertheless, by developing this algorithm, we also want to focus on the importance of these non-specific symptoms as they might be overlooked. Therefore, the algorithm suggest to perform a blood test, including NT-proBNP, and refer the patient to a cardiologist in case of doubt.

Even when NT-proBNP is not the main focus of the results, we might draw some attention on its importance. If there is a suspicion of HF, the GP could already perform a blood analysis, including the measurement of NT-proBNP levels. According to the ESC guidelines, the plasma concentration of natriuretic peptides can be used as an initial diagnostic test in patients with dyspnea to rule out the possibility of HF. For patients whose medical history or symptoms suggest HF in combination with NT-proBNP/BNP values above the upper limit, a diagnosis of HF is expected. Patients can then surely refered to a cardiologist to undergo further examination such as echocardiography [1, 21]. As the measurement of natriuretic peptides is even available as a point‐of‐care test, it can be used in routine primary care practice with minimal training. The diagnostic value of this test in patients presenting to their GP with new symptoms suggestive of HF was studied in a multi‐center study. In this study, five GP groups were asked to evaluate adult patients who suspected of having heart failure and to test NT-proBNP with a point-of care NT-proBNP device. Eighteen out of 19 GPs confirmed that the device influenced their clinical practice. During th study, GPs’ confidence in using NT-proBNP increased significantly from mean score 4.4 to 7.6 out of 10 [22]. Therefore, it is clear that more focus is needed on the use of NT-proBNP in clinical practice and even when not reimbursed, GPs should consider it in certain situations of doubt as NT-proBNP/BNP have an overall favorable diagnostic utility [23]. During the round table discussion with cardiologists to finetune the algorithm, the important value of NT-proBNP for GPs was also emphasized. Unfortunately, despite these strong benefits of NT-proBNP as a diagnostic tool, only a poor consensus was reached in our Delphi panel on the use of NT-proBNP to confirm HF. Possible reasons for this could be, from a cardiologist perspective, that there will be no real need for NT-proBNP measurement as they will rely on echocardiographic data to diagnose the patient. From a GPs’ point of view, the poor consensus can be explained by the fact that NT-proBNP is not reimbursed in Belgiumand the cost will be too high for the patient and therefore only a small minority of the GPs will actually measure it. Anyhow, we believe that more focus on the use of NT-proBNP in clinical practice is needed even when not reimbursed but GPs should consider it in certain situations of doubt. This algorithm could help GPs to define potential HF patients and suggests the use of NT-proBNP to validate this potential diagnosis.

After referral, proper diagnosis and initiation of treatment is done by the cardiologist and we suggest that the patient will be followed by his cardiologist during the following 3–6 months to make sure the patient is fully stabilized and that medication is titrated to the optimal doses. Next, it is of importance that the HF patient is managed in a cross-sectional health care process that includes all the professionals involved, such as treating cardiologist, primary care physician, nursing staff, as well as social services [24].

The Delphi panel showed no consensus on the uptitration of HF medication at the GP level, as GPs don’t feel comfortable with the disease and change in medication. Neverthless, there was a good consensus at the cardiologist level who stated that there is an important role for GPs in the uptitration of HF medication. A similar observation could be made for the downtitration of diuretics: while GPs don’t feel comfortable to downtitrate diuretics, cardiologists stated that this should be an important task for the GPs. While it has been demonstrated in several registries and surveys that there is rather a good physician’s adherence to ACEI (> 60%) and diuretics (> 80%), the adherence to beta-blockers (BB) and mineralocorticoid receptor antagonists (MRA) was much lower (30 to 60%) [25–28]. Of interest, it is clear that exclusive use of the percentage of patients treated by guideline-recommended drugs is a poor indicator of the quality of healthcare in HF. Attainment of optimal dosing in each patient should be obtained as there is evidence that higher doses of guideline-recommended drugs are associated with improved outcomes [29–32].

This study has also some limitations, as there were only 15 GPs and 7 cardiologists included in the panel, representing more than 10000 GPs and 1000 cardiologists in Belgium [33, 34]. Nevertheless, the classical Delphi method has been chosen over a general survey with a higher amount of participants, and therefore the typical panel size between 10 and 20 was used [11]. Another limitation is the rigidity of the algorithm e.g. on the referral after specific complaints. We understand that in clinical practice a referral to a cardiologist is not always going smooth regarding waiting times. An option here could be a direct phone consultation of the cardiologist by the GP. Next, one of the inclusion criteria for the recruitment of GPs in this study was an interest and knowledge of HF. Therefore we can state that the knowledge of the GPs and cardiologists is overestimated compared with the average GP in Belgium. On the other hand, this algorithm is specially created to help GPs who have less experience with HF, so therefore GPs with a certain familiarity of HF had to be included in the panel of this study for the creation of the algorithm. Finally, the selected panel were only HCPs from Belgium, therefore some bias related to Belgian clinical practice must be taken into account as this can sometimes differ as compared to other countries.

## Conclusions

Based on a Delphi panel method, we were able to create an algorithm which could help GPs in the diagnoses and follow-up of HF patients. The diagnosis should be based on three pillars, i.e. medical history, anamnesis and clinical examination that should be evaluated by the GP. If there is a suspicion of HF, the GP could already perform a blood analysis, including the measurement of NT-proBNP levels, followed by referral to the cardiologist who will be responsible for diagnosis and initiation of treatment. Afterwards, it is crucial that the HF patient is managed in a multidisciplinary health care process between the cardiologist and the GP, with an important role for the GP in uptitration of HF medication, downtitration of the dose of diuretics if possible and to assure drug compliance by the patient.

## Supporting information

S1 Fig (PDF)

S2 Fig (PDF)

S3 Fig (PDF)
