# Supplementary material for: Diagnosis, treatment, and follow-up of heart failure patients by general practitioners: A Delphi consensus statement
Source: PLoS One. 2020 Dec 31;15(12):e0244485. doi: 10.1371/journal.pone.0244485 (PMC7775077; doi:10.1371/journal.pone.0244485)
Supplement: S1 Fig — (PDF) [file pone.0244485.s001.pdf]

Questions Delphi Project

De eerste reeks vragen zijn gericht op diagnose van hartfalen (HF). De vragen zijn voorzien van een open veld, gelieve dus steeds een zo uitgebreid mogelijk antwoord te geven.

I. <u>Diagnosis</u>

1. Wat zijn volgens u de 5 belangrijkste vragen die u moet stellen tijdens anamnese bij vermoeden van HF?
2. Welke comorbiditeiten en/of karakteristieken in de voorgeschiedenis van de patient linkt u aan HF?
3. Bij welke 5 fysieke symptomen vermoedt u HF?
4. Welke klinische onderzoeken doet u bij vermoeden van HF?
5. Bij welke afwijkingen van klinische onderzoeken vermoedt u HF?
6. Welke parameters selecteert u bij een bloedonderzoek bij vermoeden van HF?

De 2de reeks vragen zijn met betrekking tot behandeling van HF patienten en de doorverwijzing naar specialisten. Ook hier zijn de vragen voorzien van open velden en zouden we willen vragen om een zo uitgebreid mogelijk antwoord te geven.

II. Treatment & Referral

7. Wat zijn voor u de belangrijkste parameters uit deel 1 waarop u uw diagnose van HF baseert en u de patient naar de cardioloog stuurt?
8. Met welke medicatie stuurt u de volgende patiënt naar huis alvorens door te verwijzen naar de cardioloog?
    a. Patiënt met kortademigheid in rust en/of inspanning
    b. Patiënt met kortademigheid (in rust/inspanning) + oedeem
    c. Patiënt met kortademigheid (in rust/inspanning) + hartproblematiek
    d. Patiënt met kortademigheid (in rust/inspanning) + longproblematiek
    e. Patiënt met kortademigheid (in rust/inspanning) + hypertensie
    f. Patiënt met kortademigheid (in rust/inspanning) + diabetes
    g. Patiënt met kortademigheid (in rust/inspanning) + slechte nierfunctie

De 3de reeks vragen betreffen de opvolging van HF patienten eens ze een cardioloog bezocht hebben. Ook dit zijn open vragen en vragen we u om een zo uitgebreid mogelijk antwoord te geven.

III. Follow up

9. Patiënt komt na zijn visite bij de cardioloog bij u en voelt zich goed.
    a. Waaruit bestaat uw controle onderzoek?
    b. Indien u medicatie aanpast, welke past u dan aan?
    c. Op basis van welke parameters past u de medicatie aan?
10. Waaruit bestaat uw controle onderzoek indien de patiënt na zijn visite bij de cardioloog bij u komt met de volgende klachten:
    a. Kortademigheid
    b. Oedeem
    c. Duizeligheid
    d. Lage bloeddruk (asymptomatisch)
    e. Lage bloeddruk (symptomatisch)
    f. Vermoeidheid
11. Welke medicatie past u aan bij een patiënt met de volgende klachten?
    a. Kortademigheid
        i. Welke bijkomende parameters analyseert u bij deze patiënt?
    b. Oedeem
        i. Welke bijkomende parameters analyseert u bij deze patiënt?
    c. Duizeligheid
        i. Welke bijkomende parameters analyseert u bij deze patiënt?
    d. Lage bloeddruk (asymptomatisch)
        i. Welke bijkomende parameters analyseert u bij deze patiënt?
    e. Lage bloeddruk (symptomatisch)
        i. Welke bijkomende parameters analyseert u bij deze patiënt?
    f. Vermoeidheid
        i. Welke bijkomende parameters analyseert u bij deze patiënt?
12. Op basis van welke parameters beslist u bloedanalyse te doen bij een gediagnosticeerde HF patiënt?
13. Hoe vaak beslist u bloedanalyse te doen bij een gediagnosticeerde HF patiënt?
14. Welke parameters selecteert u bij een bloedonderzoek bij een gediagnosticeerde HF patiënt?
