# Supplementary material for: Diagnosis, treatment, and follow-up of heart failure patients by general practitioners: A Delphi consensus statement
Source: PLoS One. 2020 Dec 31;15(12):e0244485. doi: 10.1371/journal.pone.0244485 (PMC7775077; doi:10.1371/journal.pone.0244485)
Supplement: S2 Fig — (PDF) [file pone.0244485.s002.pdf]

Questions Projet Delphi

La première série de questions se focalisent sur le diagnostic de l'insuffisance cardiaque (IC). Les questions sont pourvues de suffisamment d'espace pour répondre, veuillez donc toujours répondre de façon la plus détaillée possible.

I. Diagnostic

1. Quelles sont selon vous les 5 questions les plus importantes que vous devriez poser pendant l'anamnèse si une IC est suspectée ?
2. Quelles comorbidités et/ou caractéristiques de l'historique du patient associez-vous à l'IC ?
3. Pour quels 5 symptômes physiques suspectez-vous une IC ?
4. Quels examens cliniques faites-vous si vous suspectez une IC ?
5. A partir de quelles déviations des examens cliniques suspectez-vous une IC ?
6. Quel bilan sanguin faites-vous quand vous suspectez une IC ?

La 2e série de questions concerne le traitement des patients atteints d'IC et leur référence vers des spécialistes. Ici aussi, les questions sont pourvues de suffisamment de place afin de répondre de façon la plus complète possible.

II. Traitement et référence

7. Quels sont pour vous les paramètres les plus importants de la partie 1 sur lesquels vous basez votre diagnostic d'IC et envoyez le patient chez le cardiologue ?
8. Quels médicaments prescrivez-vous au patient décrit ci-dessous avant de référer au cardiologue?
    a. Patient souffrant de dyspnée au repos et/ou à l'effort
    b. Patient souffrant de dyspnée (au repos et/ou à l'effort) + œdème
    c. Patient souffrant de dyspnée (au repos et/ou à l'effort) + problème cardiaque
    d. Patient souffrant de dyspnée (au repos et/ou à l'effort) + problème pulmonaire
    e. Patient souffrant de dyspnée (au repos et/ou à l'effort) + hypertension
    f. Patient souffrant de dyspnée (au repos et/ou à l'effort) + diabète
    g. Patient souffrant de dyspnée (au repos et/ou à l'effort) + mauvaise fonction rénale

La troisième série de questions concerne le suivi des patients atteints d'IC après consultation chez le cardiologue. Ce sont aussi des questions ouvertes et nous vous demandons de donner une réponse aussi complète que possible.

III. <u>Follow up</u>

9. Le patient vient chez vous après sa visite chez le cardiologue et se sent bien.
    a. En quoi consiste votre consultation de contrôle?
    b. Quels médicaments ajustez-vous?
    c. Sur base de quels paramètres ajustez-vous le médicament?
10. En quoi consiste votre consultation de contrôle quand le patient vient chez vous après sa visite chez le cardiologue avec les symptômes suivants :
    a. Dyspnée
    b. Oedème
    c. Vertige
    d. Tension artérielle basse (asymptomatique)
    e. Tension artérielle basse (symptomatique)
    f. Fatigue
11. Quels médicaments ajustez-vous pour un patient présentant les symptômes suivants ?
    a. Dyspnée
        i. Sur base de quels paramètres supplémentaires?
    b. Oedème
        i. Sur base de quels paramètres supplémentaires?
    c. Vertige
        i. Sur base de quels paramètres supplémentaires?
    d. Tension artérielle basse (asymptomatique)
        i. Sur base de quels paramètres supplémentaires?
    e. Tension artérielle basse (symptomatique)
        i. Sur base de quels paramètres supplémentaires?
    f. Fatigue
        i. Sur base de quels paramètres supplémentaires?
12. Sur base de quels paramètres décidez-vous de faire un bilan sanguin sur un patient atteint d'IC diagnostiquée ?
13. A quelle fréquence décidez-vous de faire un bilan sanguin sur un patient atteint d'IC diagnostiquée ?
14. Quel bilan sanguin faites-vous sur un patient atteint d'IC diagnostiquée ?
