# Supplementary material for: Diagnosis, treatment, and follow-up of heart failure patients by general practitioners: A Delphi consensus statement
Source: PLoS One. 2020 Dec 31;15(12):e0244485. doi: 10.1371/journal.pone.0244485 (PMC7775077; doi:10.1371/journal.pone.0244485)
Supplement: S3 Fig — (PDF) [file pone.0244485.s003.pdf]

Questions Delphi Project

The first set of questions focuses on the diagnosis of heart failure (HF). The questions are provided with an open field, so please, always answer as extensive as possible.

I. Diagnosis

1. What do you think are the 5 most important questions you should ask during anamnesis when HF is suspected?
2. Which comorbidities and/or characteristics in the patient's history do you link to HF?
3. For which 5 physical symptoms do you suspect HF?
4. Which clinical investigations do you do if you suspect HF?
5. For which abnormalities of clinical examinations do you suspect HF?
6. Which parameters do you select in a blood test if HF is supected?

The 2nd series of questions are related to the treatment of HF patients and the referral to specialists. The questions are provided with open fields and therefore we would like to ask you to provide an answer as extensive as possible.

II. <u>Treatment & Referral</u>

a. What are the most important parameters from Part 1 on which you base your diagnosis of HF and send the patient to the cardiologist?
b. What medication do you prescribe for the below mentioned patients before referral to the cardiologist?
    i. Patient with dyspnea at rest and/or exercise
    ii. Patient with dyspnea (at rest/exercise) + edema
    iii. Patient with dyspnea (at rest/exercise) + heart problems
    iv. Patient with dyspnea (at rest/exercise) + lung problems
    v. Patient with dyspnea (at rest/exercise) + hypertension
    vi. Patient with dyspnea (at rest/exercise) + diabetes
    vii. Patient with dyspnea (at rest/exercise) + poor renal function

The 3rd set of questions concerns the follow-up of HF patients once they have visited a cardiologist. These questions are also open field questions and therefore, we ask you to provide an answer as detailed as possible.

III. <u>Follow up</u>

7. The patient comes to you after his visit to the cardiologist and feels well
    a. What examination do you perform?
    b. If you adjust medication, which one do you adjust?
    c. Based on which parameters do you adjust medication?
8. What does your examination consist of if the patient comes to you with the following complaints after his visit to the cardiologist:
    a. Dyspnea
    b. Edema
    c. Dizziness
    d. Low blood pressure (asymptomatic)
    e. Low blood pressure (symptomatic)
    f. Fatigue
9. Which medication do you adjust for a patient with the following complaints?
    a. Dyspnea
        i. What additional parameters are you analyzing for this patient?
    b. Edema
        i. What additional parameters are you analyzing for this patient?
    c. Dizziness
        i. What additional parameters are you analyzing for this patient?
    d. Low blood pressure (asymptomatic)
        i. What additional parameters are you analyzing for this patient?
    e. Low blood pressure (symptomatic)
        i. What additional parameters are you analyzing for this patient?
10. Based on which parameters do you decide to perform a blood analysis in a diagnosed HF patient?
11. How often do you decide to do a blood analysis on a diagnosed HF patient?
12. Which parameters do you select for a blood test on a diagnosed HF patient?
